# Epidemiology of neural tube defects and folic acid

**DOI:** 10.1186/1743-8454-1-5

**Published:** 2004-12-10

**Authors:** David B Shurtleff

**Affiliations:** 1 Department of Pediatrics, University of Washington, Seattle, WA 98105, USA

## Abstract

This review article combines four disparate observations about Neural Tube Defects (NTDs). They are the worldwide decline in the birth incidence that began prior to prenatal diagnosis; family recurrence risks; the effect of prenatal diagnosis and termination of affected pregnancies; and the effect of folic acid.

## Discussion

### Variations in birth incidence

**Figure 1:**
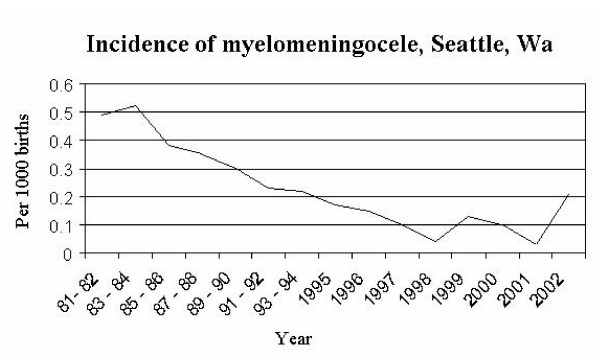
These data were obtained from the Birth Defects Clinic attendance records. Patients with myelomeningocele seen in this clinic represent 98% of individuals with myelomeningocele born in Washington State, USA, during the period that the state maintained a Birth Defects Registry. Preliminary data for the year 2003 suggest a decline in incidence to near the lowest level observed prior to 2002.

NTDs are due to many different causes [1]. The incidence varies and some etiologies are more common at different prenatal ages and birth [2-5]. Epidemiological data from the period before in-utero diagnosis and termination of affected pregnancies established two trends. The first illustrated epidemics of the incidence of NTDs that cycled over years, and the second established that there has been a general decline in the birth incidence of NTDs over several decades, which has been noted worldwide. The best example of epidemics of the occurrence of NTDs was reported from Birmingham, England [6]. The prevalence rose from 2.0 per 1000 births in 1936–7, to 2.8 in 1940, fell to 1.54 in 1948 and peaked again in 1956 at 2.8 per 1000. Elwood and Elwood [7] have documented both "epidemics" and the decline in incidence worldwide. Other "epidemics" have been described in Boston, Providence and New York in the USA, Canada, Berlin and in Western Germany, and in a number of other areas [7]. The general decline in birth incidence has been reported in most countries studied but it has occurred at different times. A variation within countries was also reported, with a Northeast to Southwest gradient from higher to lower in the UK and from East to West in Canada and the USA. For example, the highest occurrence risk for the general population on the North American continent is 2.5 – 3.5 per 1000 amongst citizens of Nova Scotia [8]. These data include findings of elective terminations, spontaneous miscarriages and births. The lowest general population occurrence is 0.21 – 0.03 per 1000 live births in the Pacific Northwest of the USA (Figure 1). These geographic variations may relate to migrations of ethnic groups. The highest birth incidences for NTDs are reported amongst descendants of the Celts in the UK, Canada and the USA. These racial data suggest the importance of a genetic component that has been borne out in family studies [7].

### Family recurrence risks

Family studies suggest the recurrence risk for first-degree relatives of affected individuals is approximately 1 in 30. For second-degree relatives (the children of the mother's sisters and brothers) the risk is approximately 1 in 220 [9]. However, the authors of the same study state there is not yet agreement upon the accurate recurrence risk data for family members. Others report a recurrence risk among the mother's or an affected child's first-degree relatives is as high as 1 in 70 to 140 [10,11]. McManus [12] reports recurrence risks in first and second-degree relatives of 1 in 40 for sisters of the mother of an affected child, and 1 in 90 for the offspring of those sisters. She reported the mother's brothers and the father's sisters and brothers to have lower recurrence risks of 1 in 140 to 1 in 190. Arata *et al *[13] report that affected mothers appear to have only a 0.5 to 1% chance of having a child with an NTD. This last observation concurs with our finding that only one mother with myelomeningocele has an affected child. That child is one of 106 otherwise unaffected by an NTD.

### Prenatal diagnosis and termination

The third aspect, prenatal diagnosis and termination of affected pregnancies, is one that should be discussed with all women in the reproductive age range and, more importantly, with patients who have a family history of an NTD. Folic acid taken orally on a daily basis is shown to lower the occurrence and recurrence of NTDs in their own offspring and in their relatives. The Medical Research Council [14] was the first to prove conclusively that when women who had had a previous child affected by an NTD took 4.0 mg of folic acid daily, beginning three months prior to conception, there was a 70% reduction in the recurrence in subsequent offspring. Wald *et al *[15] have recommended 5 mg daily. Because of the higher occurrence in first- and second-degree relatives, we recommend 4.0 mg daily, beginning three months prior to a planned conception. We suggest the effect in the United States may be nearer to a 40 – 50% reduction for two reasons. First, Berry *et al *[16] demonstrated a 79% reduction in occurrence in north China (an area of high incidence) but only 40% in south China (where there is a low incidence) when the women took 0.4 mg of folic acid periconceptually. The incidence of NTDs in the United States is closer to that seen in south China rather than that in north China. Secondly, recent data from Canada and Mexico are the first to indicate that lower incidence communities on the North American continent can achieve a 45 – 55% reduction in occurrence with a regimen of 5 mg of folic acid per week [17] and dietary fortification added to recommendations for supplementation periconceptually with 0.4 mg dose of folic acid daily [8,18].

### Folic acid supplementation

When recent trends in the birth incidence of NTDs are reported, they focus additionally on the effect that folic acid has on the early second trimester prevalence of affected fetuses. As for the epidemiological studies noted above, these reports include varying types of cases; some report only "spina bifida", others "spina bifida" and anencephaly, and still others mention these two types and encephalocele with or without hydrocephalus. Some studies report only deaths due to complications in these groups of patients as stillborns, or deaths in the neonatal time period; other reports study all affected newborns, and still others cover selective or spontaneously aborted fetuses. Those that include time intervals after the introduction of intrauterine diagnosis and selective termination do not take into consideration the variations in incidence at different gestational ages and at birth, whether stillborn or live [5]. Creasy and Alberman [2] reported that 3% of 1216 (30 per 1000) spontaneously aborted fetuses had central nervous system malformations. The majority of the malformations were NTDs. Forty percent also had chromosomal aberrations. The prevalence varied from about 21 per 1000 during each three-week period of gestational age between 8 and 19 weeks, to 105 per 1000 amongst fetuses greater than 27 weeks gestational age. The live born birth incidence at that time was 1.5 per 1000. Nishimura et al [4], reported 13 per 1000 spina bifida embryos amongst 3402 induced abortion fetuses for social reasons at a gestational age between 3 and 10 weeks old. The live born incidence at the time was 2 per 1000. Adams et al [19] reported 10 of 34 fertilized ova up to the age of 17 days were malformed (an incidence of 294 per 1000). Studies after the initiation of prenatal diagnosis and before folic acid supplementation and fortification clearly demonstrate a remarkable decrease in live born birth incidence ([1,20,21] and Figure 1). In Washington State, USA, prenatal diagnosis and termination of affected pregnancies began in 1980. Beginning in 1991, concerned specialists and the media advised supplementation with 400 mcg (0.4 mg) of folic acid daily for women in the childbearing age group. Fortification with an estimated 140 mcg (0.14 mg) per serving of flour-containing foods was added to the recommendations, beginning in 1996, and implemented over the next three years. The marked increase of 7 fold from 0.03 per 1000 in 2001 to 0.21 in 2002 coincided with the completion of fortification (Figure 1).

## Conclusions

Our data and this review clearly demonstrate the effects of intrauterine diagnosis and selective termination prior to the recommendation for supplementation and fortification of foodstuffs with folic acid. Because the reason for termination of a pregnancy is not reportable in our state and the USA, we cannot determine the effect of folic acid on the prevalence of myelomeningocele and anencephaly in first and early second trimester fetuses. Studies of the effect of folic acid in reducing the birth incidence in communities with a low incidence, and active prenatal diagnosis associated with termination of affected fetuses, require longer-term studies than published to date. The differences in data discussed above need to be considered if one is to evaluate the effect of prenatal diagnosis and elective termination as well as the effects of fortification or supplementation with folic acid. We recommend that these variables be discussed with women of reproductive age, particularly if they are relatives of a patient with an NTD. Regardless of the uncertainties, we recommend supplementation of the diet of women, beginning three months prior to an anticipated pregnancy. We recommend all women of childbearing age take at least 400 mcg of folic acid daily when they begin sexual activity. Relatives of a patient with an NTD should take 4.0 mg daily, beginning three months prior to conception.

## Competing Interests

The authors declares that he has no competing interests.
